# Germline *de novo* variants in *CSNK2B* in Chinese patients with epilepsy

**DOI:** 10.1038/s41598-019-53484-9

**Published:** 2019-11-29

**Authors:** Jinliang Li, Kai Gao, Shuying Cai, Yin Liu, Yuzhen Wang, Shaoping Huang, Jian Zha, Wenjing Hu, Shujie Yu, Zhixian Yang, Han Xie, Huifang Yan, Jingmin Wang, Ye Wu, Yuwu Jiang

**Affiliations:** 1 Department of Pediatrics, Peking University First Hospital, Beijing, 100034 China; 2 Department of Pediatric Neurology Rehabilitation, Maternal and Child Health Care of Xiamen, Xiamen, Fujian, 361003 China; 3 Department of Pediatric Neurology, Tangshan Maternal and Child Health Hospital, Tangshan, Hebei, 063000 China; 4 Department of Pediatrics, The Second Affiliated Hospital of Xi’an Jiaotong University, Xi’an, Shanxi 710004 China; 5 Department of Pediatric Neurology, Jiangxi Provincial Children’s Hospital, Nanchang, Jiangxi 330006 China; 6 Second Department of Neurology, Hunan Province Children’s Hospital, Changsha, Hunan 410007 China; 7 Department of Pediatric Neurology, Harbin Children’s Hospital, Harbin, Heilongjiang 150010 China

**Keywords:** Molecular medicine, Molecular medicine, Molecular medicine, Molecular medicine, Epilepsy

## Abstract

*CSNK2B*, which encodes the beta subunit of casein kinase II (CK2), plays an important role in neuron morphology and synaptic transmission. Variants in *CSNK2B* associated with epilepsy and/or intellectual disability (ID)/developmental delay (DD) have been reported in five cases only. Among the 816 probands suspected hereditary epilepsy whose initial report of trio-based whole exome sequencing (WES) were negative, 10 *de novo* pathogenic or likely pathogenic variants of *CSNK2B* in nine probands were identified after reanalysis of their raw Trio-WES data. Six of the nine epileptic patients had ID/DD. The age of seizure onset of these nine patients with *CSNK2B* variants ranged from 2–12 months. Eight patients had age of seizure onset of less than 6 months. The epilepsy of most probands (8/9) was generalized tonic-clonic seizure and clustered (6/9). Most patients had normal electroencephalogram (5/9) and brain magnetic resonance image (7/9) results. Most patients (7/9) had easy-to-control seizures. Levetiracetam was the most commonly used drug in seizure-free patients (5/7). The variants detected in five patients (5/9, 55.6%) were located in the zinc-binding domain. In summary, our research provided evidence that variants in *CSNK2B* are associated with epilepsy with or without ID/DD. *CSNK2B*-related epilepsy is relatively easy to be controlled. The zinc-binding domain appears to be the hotspot region for mutation.

## Introduction

Epilepsy is one of the most common diseases of the nervous system. A total of 50–100 million cases have been recorded worldwide, and 2–4 million new cases are diagnosed each year^1^. The major comorbidity of epilepsy is intellectual disability (ID)/developmental delay (DD), which affects approximately 26% of children with epilepsy^2^. The most prevalent chronic health condition in children with ID/DD is epilepsy^3,4^. Hence, epilepsy and ID/DD may share a common etiology, such as genetic factor.

With the rapid progress of next-generation sequencing, researchers have discovered an increase in the genetic causes of brain disorders, such as epilepsy, ID and autism^5,6^. Whole exome sequencing (WES) has a diagnosis rate of 25–50%^7–10^. However, 50–75% of cases have a negative diagnosis after WES. *De novo* mutations are the important causes of early-onset epileptic encephalopathies^11–14^. Trio (parents–proband) WES is better than proband-only WES^9,15^, mainly because the former can easily identify *de novo* variants and compound heterozygous variants in the proband. Additional diagnosis rates of 10–36% have been recorded after sequencing data reanalysis^16–19^.

Casein kinase 2 beta (*CSNK2B*) mutations associated with epilepsy and/or ID/DD have only been reported in five cases^20–22^. Huillard *et al*.^23^ showed that *CSNK2B* has an important role in brain development and the disruption of *Csnk2b* in mice leads to severe neurodevelopmental disorder. Cui-Ping Yang *et al*.^24^ showed that the knockdown of *CSNK2B* alters synaptic transmission and neuron morphology. In this study, we reanalysed the trio-WES data among 816 families, whose probands involve patients with epilepsy with/without ID/DD and for whom the initial genetic reports were negative. We found nine epileptic patients with pathogenic *de novo* variants of *CSNK2B* and summarised the clinical features, effective medication, and genotype-phenotype relation for *CSNK2B*-related epilepsy.

## Materials and Methods

### Patients

From June 2016 to October 2018, 816 probands suspected genetic epilepsy with initial trio-WES sequencing report were collected from Peking University First Hospital, Maternal and Child Health Care of Xiamen, Tangshan Maternal and Child Health Hospital, the Second Affiliated Hospital of Xi’an Jiaotong University, Jiangxi Provincial Children’s Hospital, Hunan Province Children’s Hospital, and Harbin Children’s Hospital. All probands were obtained from non-consanguineous families.

### Standard protocol approvals, registrations, and patient consents

This study was conducted in accordance with the Declaration of Helsinki. The protocol was approved by the Institutional Review Boards of Peking University First Hospital (2005-004). Written informed consent was obtained from all probands’ guardians.

### Variant re-analysis

We re-analysis all these trio-WES data. For each sample, exome capture was performed using xGen Exome Research Panel v1.0, which consists of 429,826 individually synthesised, and quality-controlled xGen Lockdown Probes. The captured libraries were sequenced using Illumina Novaseq. 6000 sequencer at 100× coverage. The reads were mapped to the Ensemble GRCh37/hg19 by using Burrows-Wheeler Aligner (BWA 0.7.11) with default parameters. Duplicate reads were marked with Picard Tools, version 2.9.0. Genome Analysis Tool Kit (GATK 3.5) was used for local realignment and base quality score recalibration. Variants were called jointly in all samples by using the GATK’s HaplotypeCaller. Variant Effect Predictor (VEP 93) was used to annotate variants with a predicted effect on protein-coding genes.

Variants with a sequencing depth of less than 6× and a variation frequency of less than 10% were filtered. In addition, variants were retained upon satisfying that minimum allele frequency <1% or in their absence in the 1000 Genomes Project, Exome Variant Server, or gnomAD database. Variants that were predicted to be missense, truncating, frameshift, in-frame insertion or deletion, stop codon loss, or splice site disrupting were retained. The ACMG guidelines on variant interpretation and classification were used to determine the variant’s pathogenicity^25^.

## Results

### Identification of *de novo* CSNK2B variants

**Figure 1:**
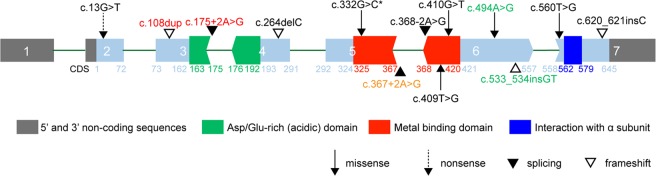
The Variants in *CSNK2B* DNA Sequence. *CSNK2B* has 648 nucleotides of coding sequence and contains seven exons. Square endings indicate that exons start or end after a complete codon, arrows to the right indicate that the last base of the last codon is located in the next exon, arrows to the left indicate that the last two bases are located in the exon. The variants in black fonts are from this study and the symbol * indicates that there are two cases with the variant. The variants highlighted in red are from recently reported patients with ID/DD and epilepsy^20,21^, the variant highlighted in gold is from recently reported patient with ID^20^, and the variants highlighted in green are from recently reported patient with profound ID and refractory epilepsy^22^.

Ten *de novo CSNK2B* variants were found in nine probands and confirmed by Sanger sequencing (Supplementary Fig. S1). All the nine probands suffered from epilepsy with or without ID/DD and had no pathogenic variation found in other epilepsy candidate genes after trio-WES reanalysis, especially *SCN1A* or *PCDH19*. The variants found in this study included c.560 T > G (p.L187R), c.620_621insC (p.F207Ffs*39), c.13 G > T (p.E5X, 211), c.256 C > T (p.R86C), c.409 T > G (p.C137G), c.264delC (p.I88Ifs*46), c.410 G > T (p.C137F), c.332 G > C (p.R111P) and c.368-2 A > G in *CSNK2B* (NM_001320) (Table 1). The variant of c.332 G > C (p.R111P) was identified in two different patients. The variants of c.13 G > T (p.E5X, 211) and c.256 C > T (p.R86C) were identified in patient 3 and were confirmed in the same allele (Supplementary Fig. S2). The variant of c.13 G > T (p.E5X, 211) resulted in a truncation after the first five amino acids. Hence, the pathogenicity of the downstream c.256 C > T (p.R86C) was negligible. The nine novel *de novo* variants found in this study and the five reported *de novo* variants are summarised in Fig. 1. The variants of the six cases occurred in the zinc-binding domain in which two were splicing variants.

**Table 1:** *In silico* analysis of variants detected in *CSNK2B*.

Case	Variant (NM_001320)	Variant origin	MAF	GERP++	CADD	SIFT	PROVEAN	PolyPhen-2	M-CAP
1	c.560 T > G (p.L187R)	*de novo*	NE	5.4	27.9	D	D	PD	D
2	c.620_621insC (p.F207Ffs*39)	*de novo*	NE	/	/	/	/	/	/
3	c.256 C > T (p.R86C)	*de novo*	NE	5.9	35	D	D	PD	D
	c.13 G > T (p.E5X,211)	*de novo*	NE	6.03	39	/	/	/	/
4	c.409 T > G (p.C137G)	*de novo*	NE	6.17	26.6	D	D	PD	D
5	c.264delC (p.I88Ifs*46)	*de novo*	NE	/	/	/	/	/	/
6	c.410 G > T (p.C137F)	*de novo*	NE	6.17	29.9	D	D	PD	D
7 and 8	c.332 G > C (p.R111P)	*de novo*	NE	5.99	32	D	D	PD	D
9	c.368-2 A > G	*de novo*	NE	4.9	23	/	/	/	/

MAF, minor allele frequency; NE, non-existent; D, damaging or deleterious; PD, probably damaging; GERP++, genomic evolutionary rate profiling; CADD, combined annotation dependent depletion.

### Predicted deleterious effect of CSNK2B *de novo* variants

*CSNK2B* is highly intolerant to variation. It has a high probability of LoF intolerance (pLI) score of 0.98 (gnomAD v2.1) and a haploinsufficiency score (%HI) of 5.78 (DECIPHER), indicating that it is extremely LoF intolerant and likely to exhibit haploinsufficiency. *CSNK2B* is constrained to mis-sense variants (*z* = 3.09; gnomAD v2.1). We used the DOMINO tool to assess the likelihood of monoallelic variants in *CSNK2B* to cause a Mendelian disease. *CSNK2B* had a DOMINO score of 0.835 out of 1, and thus monoallelic mutations likely caused the disease^26^.

**Figure 2:**
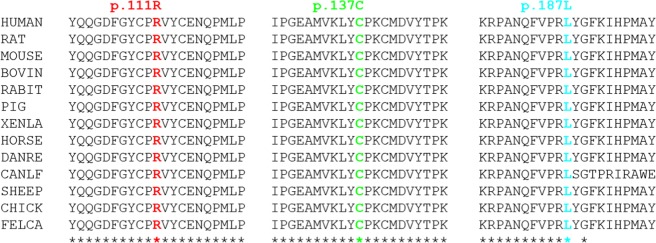
CLUSTAL O (1.2.4) multiple sequence alignment. The residues marked in color of *CSNK2B* is largely conserved across mammalian and other species. The symbol (*) under the sequences indicate identity. HUMAN, *Homo sapiens* (human); RAT, *Rattus norvegicus* (rat); MOUSE, *Mus musculus* (mouse); BOVIN, *Bos taurus* (bovine); RABIT, *Oryctolagus cuniculus* (rabbit); PIG, *Sus scrofa* (Pig); XENLA, *Xenopus laevis* (African clawed frog); HORSE, *Equus caballus* (horse); DNARE, *Danio rerio* (Zebrafish); CANLF, *Canis lupus familiaris* (dog); SHEEP, *Ovis aries* (sheep); CHICK, *Gallus gallus* (Chicken); FELCA, *Felis catus* (Cat).

All these *de novo* variants have not been found in the gnomAD and 1000 Genomes databases yet. The combined annotation-dependent depletion (CADD) scores were all very high (a score of ≥20 indicates a position within the top 1% most deleterious mutations, and a score of ≥30 indicates a position within the top 0.1% most deleterious mutations) for the variants with CADD scores^27^, indicating that these variants were deleterious. The genomic evolutionary-rate profiling (GERP)^28^ scores available for the variant site were all very high (>2 were considered constrained^29^), indicating that the nucleotide is very conservative. The amino acid residues for the variant site of patients 1, 4, 6, 7 and 8 were highly conserved across species through the sequence alignment downloaded from UniProt (Fig. 2). The pathogenicity of the identified variants was further predicted through multiple predication tools including PolyPhen2, SIFT, PROVEAN, M-CAP and MutationAssessor (Table 1). A decrease in MaxEntScan_alt score ≥ 15% compared to the MaxEntScan_ref score is considered to affect splicing^30^. The variant c.368-2 A > G in the 5th intron in the *CSNK2B* of patient 9 was a canonical splice site variation. The scores for MaxEntScan_alt (MaxEntScan alternate sequence score), MaxEntScan_ref (MaxEntScan reference sequence score) and MaxEntScan_diff (MaxEntScan score difference) were −0.946, 7.009 and 7.955, respectively, indicating that the variation was deleterious. All variants were classified as pathogenic/likely pathogenic on the basis of the guideline proposed by ACMG, which refers to the interpretations from the wInterVar web server (Table 2)^31^.

**Table 2:** Clinical interpretation of variants detected in *CSNK2B* by ACMG guideline^25^.

Case	Variant (NM_001320)	Evidence for pathogenicity based on ACMG guideline				Category
		Very strong	Strong	Moderate	Supporting	
1	c.560 T > G (p.L187R)	/	PS2	PM2	PP3 + PP4	LP
2	c.620_621insC (p.F207Ffs*39)	PVS1	PS2	PM2	PP4	P
3	c.13 G > T (p.E5X,211)	PVS1	PS2	PM2	PP4	P
4	c.409 T > G (p.C137G)	/	PS2	PM1 + PM2	PP3 + PP4	P
5	c.264delC (p.I88Ifs*46)	PVS1	PS2	PM2	PP4	P
6	c.410 G > T (p.C137F)	/	PS2	PM1 + PM2	PP3 + PP4	P
7 and 8	c.332 G > C (p.R111P)	/	PS2	PM1 + PM2	PP3 + PP4	P
9	c.368-2 A > G	PVS1	PS2	PM2	PP4	P

PVS, pathogenic very strong; PS, pathogenic strong; PM, pathogenic moderate; PP, pathogenic supporting; P, pathogenic; LP, likely pathogenic.

### Clinical features

**Figure 3:**
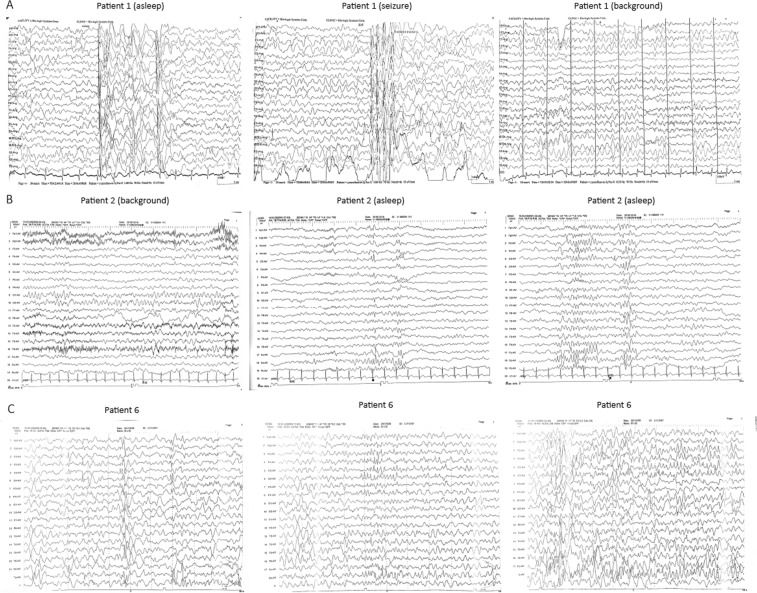
EEGs of patients with *CSNK2B* variants (patient 1, 2 and 6). (**A**) Abnormal at the age of 3.5 years with diffuse spike-slow, multiple spike-slow, and slow wave discharging. Frequent myoclonic seizures were detected. (**B**) Abnormal at the age of 3 yrs. and 5 mos. with slow background and occasionally atypical spikes in the central area on the left. (**C**) Abnormal at the age of 6 mos. with frequent sharp waves in the frontal, central, middle temporal, and middle line regions during sleep synchronously or asynchronously.

Six girls and three boys were included in this study. The seizure onset of these nine patients ranged from 2 months to 12 months. The seizure onset of eight out of nine patients was less than 6 months, indicating an early-onset epilepsy. All nine patients had epilepsy, and six of them had ID/DD. Eight of the nine patients had only one type of seizure, namely, generalised tonic-clonic seizure (GTCS). Seizures were clustered in 66.7% (6/9) patients (patients 2, 3, 4, 5, 6, and 9). We did not find any dysmorphic features in our patients, which was previously reported in two patients^21^. The brain MRIs of patients 1, 4, 5, 6, 7, 8 and 9 were normal. The EEGs of patients 3, 5, 7, 8 and 9 were normal. The EEGs of patients 1, 2 and 6 were abnormal as shown in Fig. 3. The seizures of most patients were easily controlled, and patients 3, 4, 5, 6, 7, 8 and 9 became seizure free. Levetiracetam (LEV) was the most commonly used drug among seizure-free patients (patients 3, 4, 5, 7 and 8). Four patients became seizure free after monotherapy (patient 5 and 8 with LEV, patient 6 with Valproate [VPA] and patient 9 with topiramate [TPM]). Three patients became seizure free after polytherapy. Patients 3 and 4 became seizure free with LEV and oxcarbazepine (OXC). Patient 7 became seizure free by using TPM, OXC and LEV. Three of the seven seizure-free patients had normal intelligence or developmental milestone, but the other four patients had comorbid ID/DD. Their ID/DD did not improve even after an average of nine seizure-free months (2–15 months). One of the nine patients in this study and two patients reported in a previous study showed myoclonic epilepsy; two of these patients had refractory epilepsy, and another one had epilepsy controlled upon VPA and LEV treatment. The clinical features, electroencephalogram (EEG) and brain magnetic resonance (MRI) of patients with *CSNK2B* variants are summarised in Table 3.

**Table 3:** Summary of Clinical Features.

Case number	1	2	3	4	5	6	7	8	9
Gender	Male	Female	Female	Female	Male	Female	Male	Female	Female
Age at last follow-up	6 yrs. 2 mos.	3 yrs. 5 mos.	17 mos.	14 mos.	1 yr. 8 mos.	2 yrs.	2 yrs.	1 yrs. 3 mos.	6 mos.
Perinatal history	(−)	(−)	(−)	(−)	(−)	(−)	(−)	(−)	(−)
Height (cm)	105 (<3^rd^)	92 (3^rd^–25^th^)	79 (25–50^th^)	76 (25–50^th^)	82 (25–50^th^)	80 (<3^th^)	95 (50–75^th^)	78 (25–50^th^)	NA
Weight (kg)	18 (50^th^) (3^rd^–25^th^)	15 (25–50^th^)	11 (50–75^th^)	11.5 (50–75^th^)	12 (50–75^th^)	10 (3^rd^–25^th^)	10.5 (3^rd^–25^th^)	11 (50–75^th^)	8.5 (50–75^th^)
Head circumference (cm)	48 (3.6 yrs) (3^rd^–25^th^)	49 (50^th^)	47 (50–75^th^)	46 (50–75^th^)	48 (50–75^th^)	NA	NA	45 (25–50^th^)	NA
Gross motor development	Sitting without support at 1.5 yrs; still cannot walk	Cannot jump with both feet; walking without help at 16.5 mos.	Rolling over at 4 mos.; sitting without help at 1 yr.; still cannot walk without help	Normal	Normal	Normal	Still cannot walk without help	Sitting at 7 mos.; cannot walk without help	Rolling over at 4 mos.; still cannot sit
Development of speech and language skills	Not acquired	Speak Mom and Dad at 10 mos.; say five to six words at 3 yrs.	Not acquired	Normal	Normal	Normal	Speaking only several words	Cannot speak Mom and Dad	None
ID/DD	Profound	Mild	Moderate	None	None	None	Mild	Mild	Mild
Age at seizure onset	1 yr.	4 mos.	5 mos.	2 mos.	6 mos.	6 mos.	5 mos.	5 mos.	3.5 mos.
Seizure types	Myoclonus, only twice of GTCS	GTCS	GTCS	GTCS	GTCS	GTCS	GTCS	GTCS	GTCS
Regression of development after seizure onset	None	None	None	None	None	None	None	None	None
EEG	Diffuse spike-slow, multiple spike-slow, and slow wave discharging; frequent myoclonic seizures	Normal at seizure onset; slow background and occasionally atypical spikes	Normal	Epileptiform discharge in the right anterior temporal and middle temporal regions	Normal	Sharp waves in the frontal, central, middle temporal, and middle line regions	Normal	Normal	Normal
MRI	Normal	Slightly widen of subarachnoid space at 7 mos.	Poor myelination at 5 mos.	Normal	Normal	Normal	Normal	Normal	Normal

ID/DD, intellectual disability/developmental delay; NA, not available; GTCS, generalized tonic-clonic seizure; CZP, clonazepam; LEV, levetiracetam; OXC, oxcarbazepine; VPA, valproate; OXC, oxcarbazepine; EEG, electroencephalogram; MRI, brain magnetic resonance.

Patient 1 had refractory epilepsy and profound ID with an age of seizure onset of 1 year. He is now 6 years and 2 months old, but he cannot utter the word ‘Mom’ or ‘Dad’, and he cannot sit properly. He suffered from severe myoclonic epilepsy, which occurs from dozens to 100 times a day. Only two events of GTCS were observed. The frequency of seizure increased with fever. The EEGs were abnormal at 3 years and 6 months of age with a diffuse slow spike, multiple spike-slow and slow wave discharge. Brain MRI, metabolic assays, array-based Comparative Genomic Hybridization (aCGH), chromosomal karyotype analysis, and mitochondrial DNA analysis were all negative. Multiplex ligation-dependent probe amplification (MLPA) was applied to detect copy number variation (CNV) in *SCN1A* and the result was negative (Supplementary Fig. S3A). We carefully analysed myoclonic epilepsy-related genes by using trio-based WES data, and no pathogenic variation was found (Supplementary Table S1). VPA and clonazepam were initially used for antiepileptic therapy. The antiepileptic drugs (AEDs) currently used are LEV and TPM. However, epilepsy and ID are not alleviated.

Patient 2 had epilepsy and mild DD. Her Gesell developmental quotient scores were 75 and 70 at 2 years and 2 months and at 3 years and 1 month of age. The type of seizure was GTCS. Seizures occurred four to six times daily for the first three days at the time of epilepsy onset (4 months of age). Each seizure lasted for approximately 30 s and was accompanied by fever. Then, seizures became much less frequent. The patient’s parents did not agree with the use of any AED because the parents were able to tolerate the seizures, which occurred at intervals of several months. The longest interval was 14 months. However, the seizures persisted, which were mostly accompanied by fever. The EEGs were normal at 4 months and 25 days of age and abnormal at 3 years and 5 months of age with slow background and occasionally atypical spikes in the central area on the left. Brain MRI had slightly widened subarachnoid space at 7 months of age and was normal at 22 months of age. MLPA test showed negative *SCN1A* CNV result (Supplementary Fig. S3B).

Patient 3 was a 17-month-old girl with epilepsy and mild DD. Her age of seizure onset was 5 months, and it was presented as clustered seizures with a frequency of five times in three days, each lasting for approximately 1 minute. From six to eight months of age, seizure occurred only once, but clustered seizures re-occurred after nine months, each lasting for approximately 1–3 min. Epilepsy was controlled for 3 months until the present time with LEV and OXC treatment. The patient’s EEG was normal at the age of 9 and 10 months. The brain MRI showed delayed myelination at the age of five months.

The mutated amino acids of CSNK2B in patients 4 and 6 were the same. However, the amino acid changed from cystine to glycine (c.409 T > G, p.C137G) in patient 4 and from cystine to phenylalanine (c.410 G > T, p.C137F) in patient 6. The ages of seizure onset in patients 4 and 6 were two and six months, respectively. Both patients had clustered seizures, and their seizure type was GTCS. Within the first month of seizure onset, more than 10 episodes of seizures were observed, each lasting for 1–2 min. They did not have developmental delay. Their interictal EEGs were abnormal and similar (Table 3). Their brain MRI results were normal. The seizures in patients 4 and 6 were controlled with monotherapy or polytherapy.

Patient 5 was a boy with the age of 1 year and 8 months, having an epilepsy but without ID/DD. The age of seizure onset was 6 months, and seizures occurred more than 10 times in the first month. The seizure type was GTCS. Seizure has been controlled for 1 year and 3 months until the present time.

Patient 8 was a 16-month-old girl with epilepsy and mild DD. The seizure type was GTCS, and half of the seizures were accompanied by fever. Epilepsy was under control for seven months with LEV treatment. MLPA test showed a negative *SCN1A* CNV result (Supplementary Fig. S3C). Patient 7 had the same variant and similar clinical features as patient 8, except for the absence of fever during seizures. His seizure had been controlled for 14 months with polytherapy.

In this study, only patient 9 had a splicing variant in *CSNK2B*. She is now 6 months old and has mild DD. The age of seizure onset was 3.5 months. The seizure type was GTCS, which was presented as clustered seizures, with a frequency of three times in 10 days, each lasting for 3–5 min. Seizure was controlled for more than two months.

## Discussion

*CSNK2B* encodes the beta subunit of CK2, a ubiquitous protein kinase that regulates signal transduction, metabolic pathways, replication, transcription and translation. CK2 consists of three subunits, alpha, beta and alpha prime, which form a tetrameric holoenzyme. The alpha and alpha prime subunits are catalytic and the beta subunit has a regulatory functions.

*CSNK2B* splice site mutations has been reported in two patients with ID with or without myoclonic epilepsy^20^. The patient with myoclonic seizures (c.175 + 2 A > G) had refractory epilepsy, which occurred at the age of 18 months, and no remarkable improvement was found after LEV and VPA treatment. Then, a study reported a truncating mutation in *CSNK2B* (c.108dup) that causes myoclonic epilepsy and ID^21^. Antiepileptic treatment with VPA and LEV cured both epileptic myoclonus and complex partial seizures at the age of 15 months. The brain MRIs of these three patients in a previous report were all normal. Recently, another study reported two severe patients^22^, who both had seizures at the age of 2 months, which were refractory to polytherapy. One of the two patients developed a Lennox–Gastaut syndrome and GTCS from facial clonic seizures at the age of nine years. The other patient presented focal seizures on the 5th day after birth with a frequency of two or three times per day at the age of 7 years. Both patients had severe psychomotor impairment, were bedridden, and were not able to speak any meaningful words.

The relationship between the mutations in *CSNK2B* and related disease has not yet been cited in the OMIM database possibly due to the small number of cases. We presented nine cases with *de novo* variants in *CSNK2B*. These subjects, whether with a missense, frameshift, nonsense, or splicing variant, all had seizures. Six of them also had ID/DD. Bioinformatics analysis supported the deleterious effect in *CSNK2B* at the gene and variant levels, and all the variants are classified into pathogenic or likely pathogenic on the basis of the guideline proposed by ACMG. The age of seizure onset of all nine probands was less than 12 months, and the eight probands were below the age of six months. Eight out of nine (88.9%) cases had GTCS as the only seizure type. Cluster seizures occurred in 66.7% (6/9) patients. Most cases had normal EEG (5/9) and brain MRI (7/9). Seven patients (7/9, 77.8%) became seizure free with monotherapy or combination therapy. Only one patient did not undergo any treatment, because her parents were able to control her infrequent and non-severe seizures. Her seizures were recurrent from four months after birth until the age of three years and 5 months. Her EEG changed from normal to abnormal. One of the nine patients in this study and two patients reported in a previous study showed myoclonic epilepsy. Two of them had refractory epilepsy, while the other had an epilepsy controlled via VPA and LEV treatment. LEV is the most commonly used drug in seizure-free patients (5/7). Four patients became seizure free after monotherapy (two patients with LEV, and the two other patients with VPA and TPM). Three patients became seizure free with polytherapy (two patients with LEV and OXC, the another with TPM, OXC and LEV). Thus, *CSNK2B*-related epilepsy mostly had an early onset, and GTCS was the most common seizure type. Seizures are often clustered and could be recurrent for a long time if left untreated with AEDs, although seizures are easily controlled. Myoclonic seizures might predict AED resistance.

Three of the seven seizure-free patients had normal intelligence or developmental milestone. No improvement was observed in the intelligence and development of the four other patients with ID/DD after being seizure-free for an average of nine months (in the range 2–15 months). This finding suggests that ID/DD is a common comorbidity of *CSNK2B*-related epilepsy and will persist even if seizures have been controlled. CK2 is much more abundant in the brain than in any other tissue^32^. The number of dendrites, dendritic length and branch points are remarkably reduced in neurons after *CSNK2B* knockdown; moreover, CSNK2B in neurons remarkably alters the amplitude of miniature inhibitory postsynaptic currents and the frequency of miniature excitatory postsynaptic currents, thereby suggesting that CSNK2B is required for normal synaptic transmission^24^. Recently, Huillard *et al*.^23^ found that conditional knockout of *csnk2b* in mice, lead to defects in the differentiation of embryonic neural stem cells. Moreover, Csnk2b positively regulates the development of oligodendrocyte precursor cells by interacting with transcription factor Olig2. *CSNK2B* plays an important role in brain development. However, its function in neurons remains poorly understood, and a detailed mode for its regulation has not been established. This finding might explain why ID/DD is the common co-morbidity in *CSNK2B*-related epilepsy.

Spatio-temporal expression pattern analysis in developing prefrontal cortex shows that the mRNA expression levels of *CSNK2B* were higher at the early developmental stage (i.e., from eight post-conception weeks to four months of age) than at childhood and adulthood stages^24^. The early onset of *CSNK2B*-related epilepsy may be related to the high expression at the early stage of infancy.

Notably, PCDH19-related epilepsy has similar characteristics as CSNK2B-related epilepsy^33^, such as clustered seizures and early onset (<1 year) and is often associated with ID. PCDH19-related epilepsy is characterised by fever sensitivity. CSNK2B-related epilepsy had some seizures occurred during febrile disease, but, unlike PCDH19-related epilepsy, the seizures does not have significant fever sensitivity. PCDH19-related epilepsy was X-linked inherited in which patients are mainly females with heterozygous variants, or very few males with somatic mosaic mutations. CSNK2B-related epilepsy was autosomal dominant inherited, and both men and women were equally affected.

*CSNK2B* has several domains, such as Asp/Glu-rich (acidic), zinc-binding and interaction with α subunit domains. Six variants, including the reported variant c.367 + 2 A > G, are located in the zinc-binding domain, which might be important and hotspot domains related to this type of epilepsy. However, all the five patients with variants in the zinc-binding domain became seizure free. Hence, the seizures of the patients with variants in the domain of zinc-binding were easily controlled. The variant of patient 1 was next to the domain of interaction with α subunit, and the patient with a splicing variant in the acidic domain had refractory myoclonic epilepsy. Hence, the patients with variants related to interaction with α subunit and acidic domains had severe and refractory epilepsy. However, a severe phenotype could occur in patients with missense (patient 1 and reported c.494 A > G), frameshift (reported c.533_534insGT) or splice variation (reported c.175 + 2 A > G). The mild phenotype could occur in patients with missense, splice, frameshift, or nonsense variation. Therefore, we cannot establish a definite correlation between the phenotype and genotype in *CSNK2B*-related epilepsy at this time.

In conclusion, our research further demonstrated that pathogenic variants in *CSNK2B* were associated with epilepsy with or without ID/DD. *CSNK2B*-related epilepsy always starts early, and GTCS is the most common seizure type. In this type of epilepsy, seizures may recur over time if without treatment, and were often cluster, but these seizures were always easy to control, except that myoclonic seizures were refractory. LEV is the preferred AED. The zinc binding domain is the hotspot region for mutation.

## Supplementary information

Germline de novo variants in CSNK2B in Chinese patients with epilepsy
