# Supplementary material for: Germline de novo variants in CSNK2B in Chinese patients with epilepsy
Source: Sci Rep. 2019 Nov 29;9:17909. doi: 10.1038/s41598-019-53484-9 (PMC6884442; doi:10.1038/s41598-019-53484-9)
Supplement: Supplementary file 1 — Germline de novo variants in CSNK2B in Chinese patients with epilepsy [file 41598_2019_53484_MOESM1_ESM.pdf]

Germline *de novo* variants in *CSNK2B* in Chinese patients with epilepsy

Jinliang Li[1], Kai Gao[1], Shuying Cai[2], Yin Liu[3], Yuzhen Wang[3], Shaoping Huang[4], Jian Zha[5], Wenjing Hu[6], Shujie Yu[7], Zhixian Yang[1], Han Xie[1], Huifang Yan[1], Jingmin Wang[1], Ye Wu[1], Yuwu Jiang[1,*]

[1]Department of Pediatrics, Peking University First Hospital, Beijing, China 100034

[2]Department of Pediatric Neurology Rehabilitation, Maternal and Child Health Care of Xiamen, Xiamen, Fujian, China 361003

[3]Department of Pediatric Neurology, Tangshan Maternal and Child Health Hospital, Tangshan, Hebei, China 063000

[4]Department of Pediatrics, The Second Affiliated Hospital of Xi'an Jiaotong University, Xi'an, Shanxi, China 710004

[5]Department of Pediatric Neurology, Jiangxi Provincial Children's Hospital, Nanchang, Jiangxi, China 330006

[6]Second Department of Neurology, Hunan Province Children's Hospital, Changsha, Hunan, China 410007

[7]Department of Pediatric Neurology, Harbin Children's Hospital, Harbin, Heilongjiang, China 150010

*Correspondence author: Dr Yuwu Jiang, Department of Pediatrics, Peking University First Hospital, Beijing, China 100034; Tel: +8613701398992; Fax: 010-66530532; E-mail: jiangyuwu@bjmu.edu.cn

Supplementary material

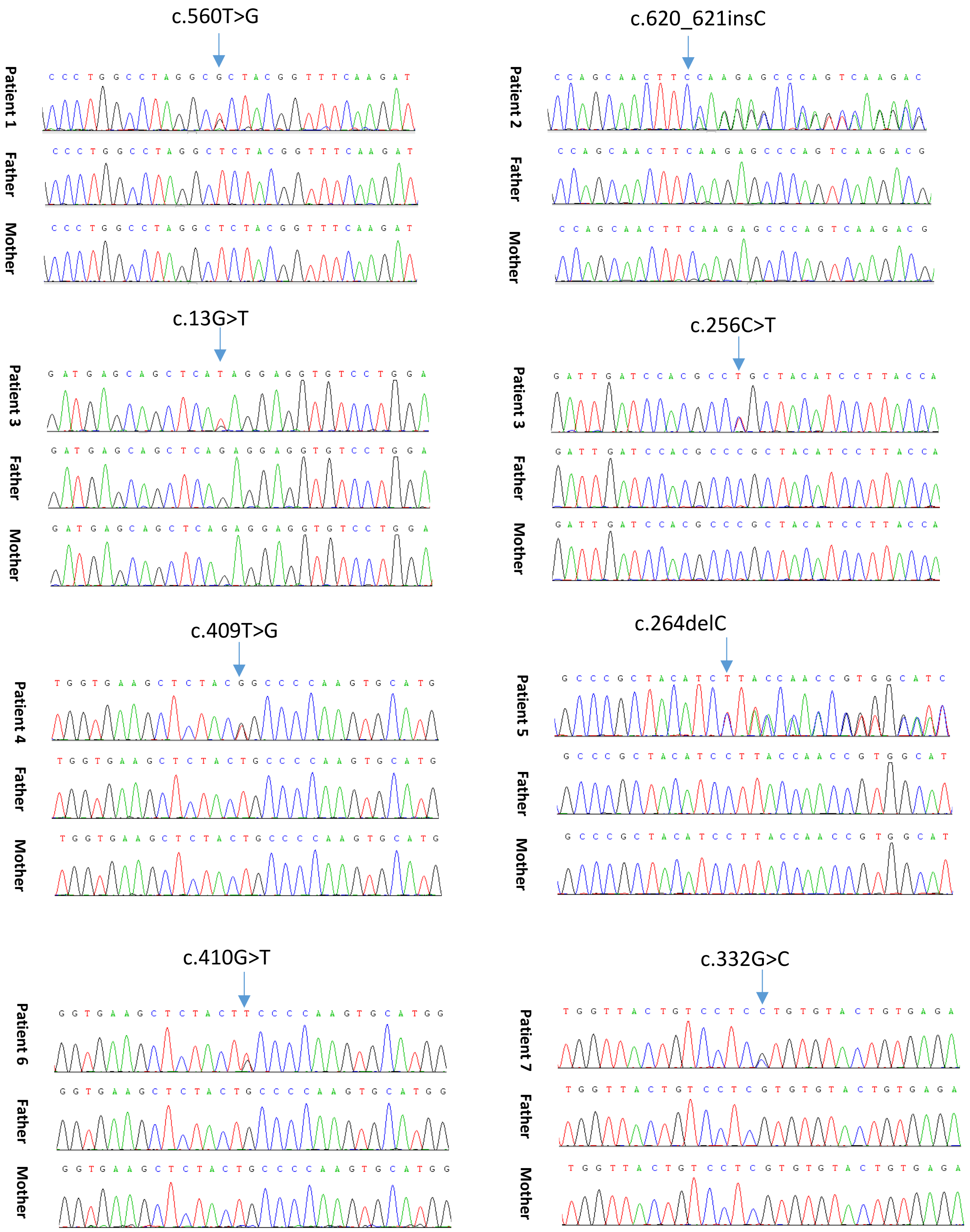

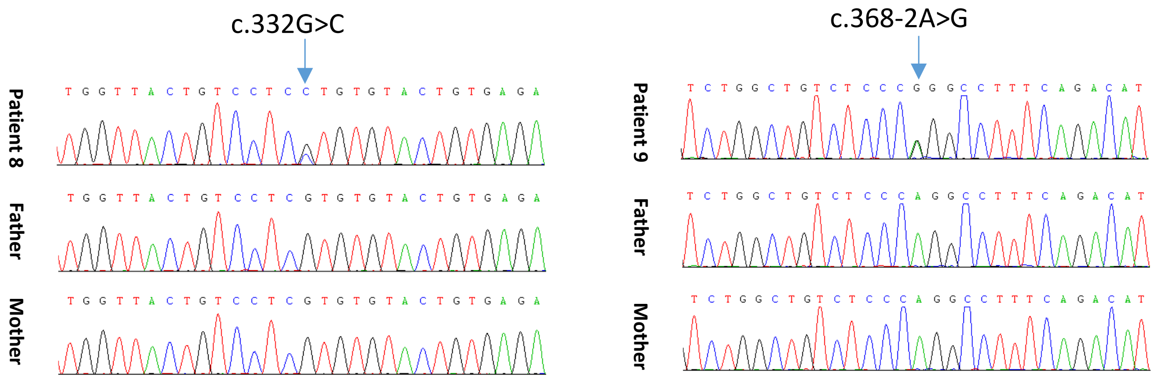

Figure S1. All the *de novo* variants in this study were confirmed by Sanger sequencing method

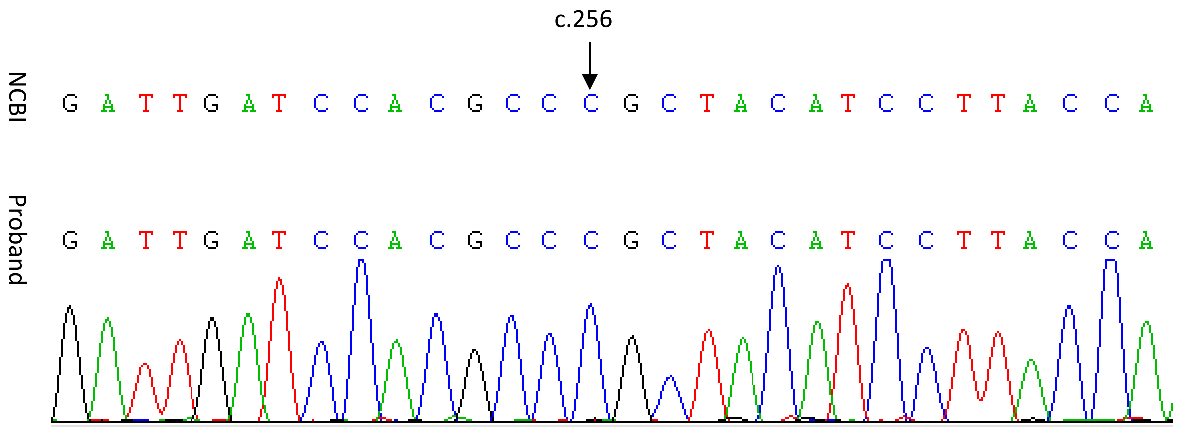

Figure S2. c.13G>T p.E5X,211 and c.256C>T p.R86C in *CSNK2B* of patient 3 were in the same allele. We identified *de novo* c.13G>T and c.256C>T in *CSNK2B* in patient 3 through whole exome sequencing (WES) and Sanger sequencing. We designed specific primers to amplify the chromatid fragment of c.13G and detected c.256 site by sequencing. The nucleotide of c.256 of the proband was C. The two *de novo* mutations c.13G>T and c.256C>T should be in the same allele.

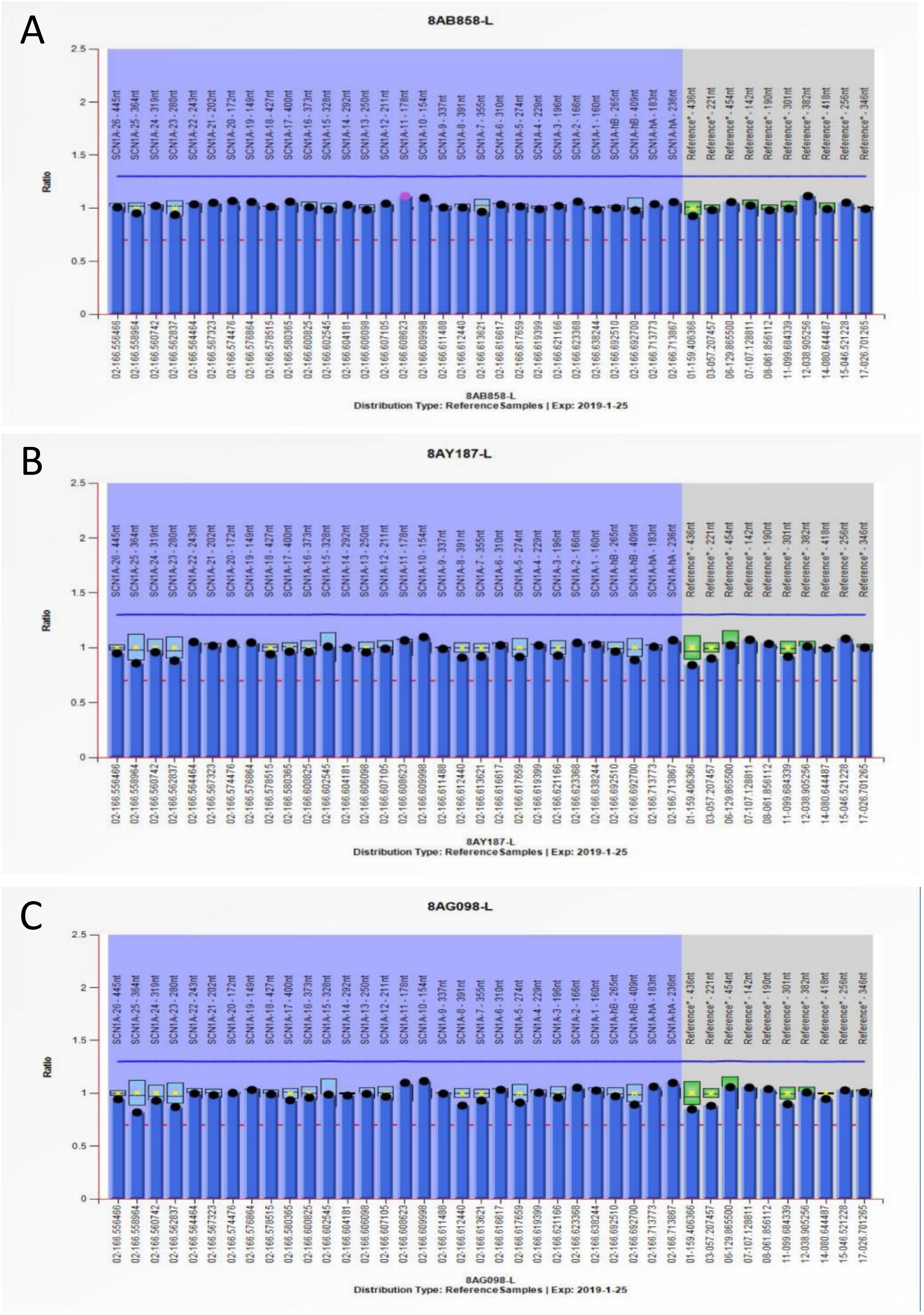

Figure S3. Multiplex ligation-dependent probe amplification (MLPA) were applied to detect copy number variation in *SCN1A* of patient 1, 2 and 8. The results of patient 1 (A), 2 (B) and 8 (C) are all negative.

Table S1. Myoclonic epilepsy related genes

Phenotype	Phenotype MIM number	Gene symbol	Inheritance
Ceroid Lipofuscinosis, Neuronal, 1; CLN1	256730	*PPT1*	AR
Ceroid Lipofuscinosis, Neuronal, 2; CLN2	204500	*TPP1*	AR
Ceroid Lipofuscinosis, Neuronal, 3; CLN3	204200	*CLN3*	AR
Ceroid Lipofuscinosis, Neuronal, 4, Parry type; CLN4B	162350	*DNAJC5*	AD
Ceroid Lipofuscinosis, Neuronal, 5; CLN5	256731	*CLN5*	AR
Ceroid Lipofuscinosis, Neuronal, 6; CLN6	601780	*CLN6*	AR
Ceroid Lipofuscinosis, Neuronal, 7; CLN7	610951	*MFSD8*	AR
Ceroid Lipofuscinosis, Neuronal, 8; CLN8	600143	*CLN8*	AR
Ceroid lipofuscinosis, neuronal, 10; CLN10	610127	*CTSD*	AR
Ceroid lipofuscinosis, neuronal, 11; CLN11	614706	*GRN*	AR
Ceroid Lipofuscinosis, Neuronal, 13; CLN13	615362	*CLN13*	AR
Unverricht and Lundborg	254800	*CSTB*	AR
Dentatorubro-pallidoluysian Atrophy, DRPLA	125370	*ATN1*	AD
Type 1 sialidosis	256550	*NEU1*	AR
Gaucher disease type 3; GD III	231000	*GBA*	AR
Spinal muscular atrophy with progressive myoclonic epilepsy; SMAPME	159950	*ASAH1*	AR
Epilepsy, progressive myoclonic 1B; EPM1B	612437	*PRICKLE1*	AR
Epilepsy, progressive myoclonic 2A/2B (Lafora); EPM2A/EPM2B	254780	*NHLRC1/ EPM2A*	AR
Epilepsy, progressive myoclonic 3, with or without intracellular inclusions; EPM3	611726	*KCTD7*	AR
Epilepsy, progressive myoclonic 4, with or without renal failure; EPM4	254900	*SCARB2*	AR
Epilepsy, progressive myoclonic 6; EPM6	614018	*GOSR2*	AR
Epilepsy, progressive myoclonic 7; EPM7	616187	*KCNC1*	AD
?Epilepsy, progressive myoclonic, 8; EPM8	616230	*CERS1*	AR
?Epilepsy, progressive myoclonic, 9; EPM9	616540	*LMNB2*	AR
?Epilepsy, progressive myoclonic, 10; EPM10	616640	*PRDM8*	AR
Myoclonic Epilepsy Associated with Ragged-Red Fibers, MERRF	545000	Mitochondrial DNA or *MTTK*	/
